# Technical Parameters for Laser Acupuncture to Elicit Peripheral and Central Effects: State-of-the-Art and Short Guidelines Based on Results from the Medical University of Graz, the German Academy of Acupuncture, and the Scientific Literature

**DOI:** 10.1155/2012/697096

**Published:** 2012-04-29

**Authors:** Gerhard Litscher, Gerhard Opitz

**Affiliations:** ^1^The Stronach Research Unit for Complementary and Integrative Laser Medicine, Research Unit of Biomedical Engineering in Anaesthesia and Intensive Care Medicine, and TCM Research Center Graz, Medical University of Graz, Auenbruggerplatz 29, 8036 Graz, Austria; ^2^German Academy of Acupuncture, 81679 Munich, Germany

## Abstract

The scientific literature in the area of laser acupuncture is rather large; however, the actual mechanisms and effects have not yet been proven in detail. Since the early days of laser acupuncture, there are still many open questions concerning technical parameters of this innovative technique. In this paper, we report about the most important technical parameters (wavelength, output power, power density, energy density, dose range, and continuous or pulsed laser) for laser acupuncture and present quantitative results for optimal laser stimulation, which allow eliciting reproducible effects in the periphery and in the brain. There are several position statements on laser acupuncture and also several review articles in scientific literature concerning clinical effectiveness of laser acupuncture. For example, the Australian Medical Acupuncture College stated recently that “the optimal energy density for laser acupuncture and biostimulation, based on current clinical experience, is 4 J/cm^2^”. However, our results of previous research studies and of this paper clearly show that dose must be adjusted according to the individual responses.

## 1. Introduction

In the Western world, it is less known that one of the medical pioneers of laser acupuncture comes from China. The surgeon Zhou used laser acupuncture in China as a type of controlled anesthetic method for dental indications since 1979 [1].

Zhou developed interesting techniques which involve irradiation of different acupuncture points. For extractions in the lower jaw, one single acupuncture point (Hegu; LI4) was irradiated for five minutes with a helium-neon laser equipment using a laser beam of 2.8–6 mW focused to a red spot on the acupuncture point [2]. The Chinese oral surgeon Zhou also works with a CO_2_ laser within the laser therapy range of 0–100 mW, which he considered already at that time more effective than the other one. Zhou performed more than 10000 tooth extractions with this laser acupuncture anesthesia. Even though it was a Chinese doctor who pioneered laser acupuncture, it was a Canadian, Friedrich Plog, who pointed out the usefulness of laser acupuncture in this context in the Western world. He was already testing lasers instead of needle acupuncture in 1973 [2, 3].

The scientific literature in the area of laser acupuncture is rather large; however, the actual mechanisms and effects have not yet been proven in detail. In the scientific database PubMed (http://www.pubmed.gov/), more than 560 referenced publications can be found at the moment (February 2012). Recent studies using modern biomedical equipment comparing the effects of laser and needle acupuncture have contributed to a better understanding and have clearly shown that laser light can be successfully used for effective acupuncture treatment. However, since the early days of laser acupuncture there are still many open questions concerning technical parameters of this innovative technique.

Within this publication, only a few aspects concerning the main technical parameters should be presented. It has to be mentioned that there are several renowned associations offering recommendations for laser therapy. For example, the “World Association for Laser Therapy” (WALT) suggests dosages between 2 and 16 Joules for laser treatment [4, 5]. In the following, we will briefly describe the most important technical parameters for laser acupuncture and present for the first time quantitative results for optimal laser stimulation in acupuncture research, which allow eliciting reproducible effects in the periphery and in the brain.

## 2. Technical Parameters for Laser Acupuncture

The following technical parameters can significantly affect the effects of laser acupuncture treatment. In addition, there are several biological parameters which depend on the subject to be investigated. The latter influences should be discussed in another publication.

### 2.1. Wavelength

**Figure 1:**
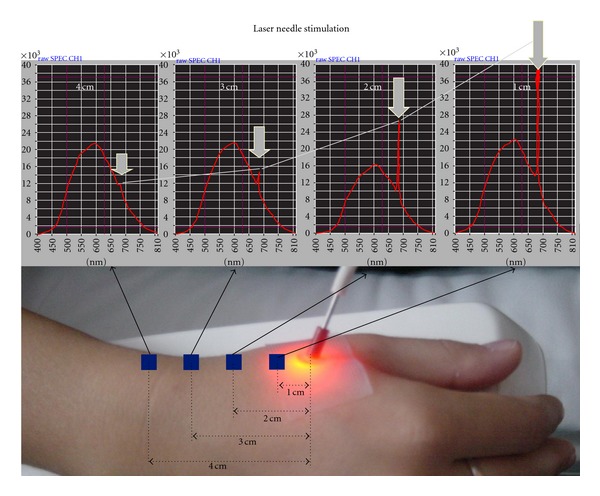
Light dispersion on the human skin. Note the peak in the spectrum at 685 nm (modified from [6]).

The question “which wavelength should be used in laser acupuncture” is sometimes related to the question “how deep does light penetrate human tissue”. It is well known that red laser light has a deeper penetration depth than violet, blue, green, or yellow. Infrared light is not visible, but some authors have demonstrated that it penetrates human tissue at least as deep as visible red light. One of our own experiments using red light (685 nm) is shown in Figure 1 [6].

Light dispersion on the skin was measured using a multiparametric device (O2C Oxygen to see, LEA Medical Technology, Gießen, Germany). Figure 1 shows that even at a distance of 4 cm the red laser light from a so-called laser needle (wavelength 685 nm, output power 40 mW, and diameter 500 *μ*m) [6] can be detected. One can conclude from this experiment that the penetration depth of red laser light with the aforementioned parameters is at least 4 cm. This is in accordance with experiments from other research groups [7]. In this example, we used a wavelength of 685 nm, as already mentioned. Other authors believe that wavelengths between 633–670 nm are the best option for laser therapy (e.g., nerve regeneration) [2]. They also describe the penetration of light of this wavelength range to be only up to one centimeter. It should be mentioned critically that any wavelength in combination with a reasonable dose at the acupuncture point may have a biological effect. Probably other parameters like the dosage may be just as important as the wavelength. On the other hand, the dosage is sometimes not known or obtainable, for example, due to the lack of penetration [2]. In some of our “laser needle” studies [6] we have shown that good experimental and clinical results can also be obtained when two wavelengths are combined. These so-called “bichromatic” laser needles were used in several previous studies [6].

### 2.2. Output Power

In order to calculate the dose to be administered at the acupoint, it is important to know the output power of the laser acupuncture instrument. Higher output power results in a higher power density, and it is also important with respect to light penetration in tissue [2]. If the acupuncture laser does not only have a continuous wave mode, but also a pulsed mode, the average output power of the laser is also important. With the average output power it is also possible to calculate the dose to administer by the pulsed laser.

### 2.3. Power Density

When using acupuncture point treatment, one must make sure that the treatment time is not too long. The parameter power density reflects the intensity of the laser beam. Its units are watts or milliwatts per cm².

### 2.4. Energy Density

The energy density is measured in watt-seconds per cm² (= Joules per cm²). Energy density is the same as dose or treatment dose. Dosage refers to the amount of energy per unit area brought to bear on tissue or cell culture [2].

### 2.5. Dose Range

The dose ranges used for laser acupuncture stimulation differ in the literature, from 0.001 J/cm² to 10 J/cm² and more. Tunér and Hode stated that “dose is a very complicated issue. It is a matter of wavelength, power density, type of tissue, condition of the tissue, chronic or acute problem, pigmentation, treatment technique, and so forth” [2].

### 2.6. Continuous or Pulsed Laser

Laser beams can be presented pulsed or continuously (see also Section 2.2). The pulsing of the laser light may interfere with other pulsing biological phenomena. This may probably have special effects, but very little is known about it today [2].

## 3. Results

### 3.1. Minimal Dose

**Figure 2:**
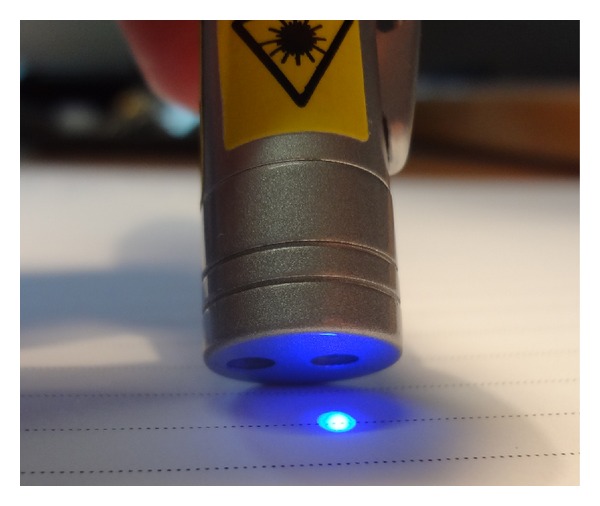
Violet laser stimulation with very low output power (1 mW). The beam area was about 0.5 cm², resulting in a power density of about 2 mW/cm² [8].

We have shown in ultralow-level laser acupuncture stimulation in rats recently that a very low power density (about 2 mW/cm²) of a violet laser beam (wavelength 405 nm, output power 1 mW, beam area ~0.5 cm², and duration 2 min) at the Baihui (GV20) acupuncture point can reproducibly modulate neurovegetative parameters (Figure 2) [8].

Significant biological effects using wavelengths of 633 or 670 nm at extremely low power densities (about 0.15 mW/cm²) were recently described also by other authors [9].

### 3.2. Optimal Dose

**Figure 3:**
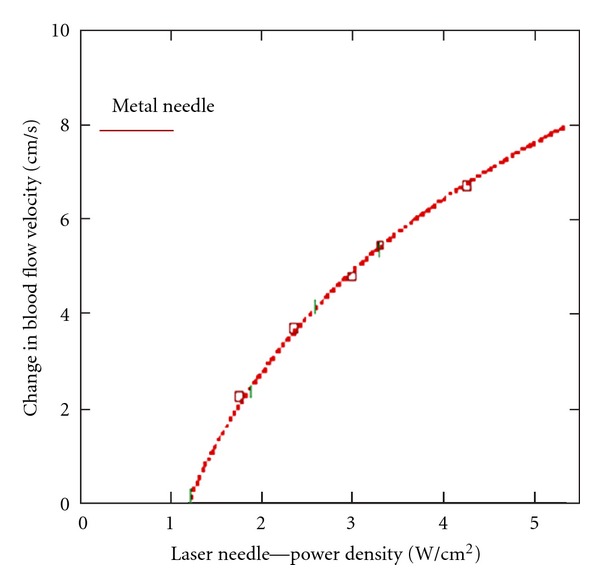
Change in blood flow velocity in the ophthalmic artery in dependence on the power density of laser needles during stimulation of an eye-specific acupuncture scheme. The mean changes measured in metal needle acupuncture are marked with a line (modified from [10]).

Concerning this topic, own results from the Medical University of Graz can be presented [10].  Figure 3 shows the detected dependency of blood flow velocity in the human ophthalmic artery as a function of power density from laser needles.

Acupuncture of seven eye-specific acupoints leads to a significant increase in blood flow velocity in the ophthalmic artery. Metal needles yield an increase from 10 cm/s to 18 cm/s [10].

It is obvious that changes in blood flow velocity are dependent upon the optical power densities applied when using laser needle acupuncture. The curve conveys the best analytical adaptation of measurement values. This curve satisfies the mathematical function  *f*(*x*) = *c* × ln⁡⁡(*x* + 0.5) [6, 10].

**Figure 4:**
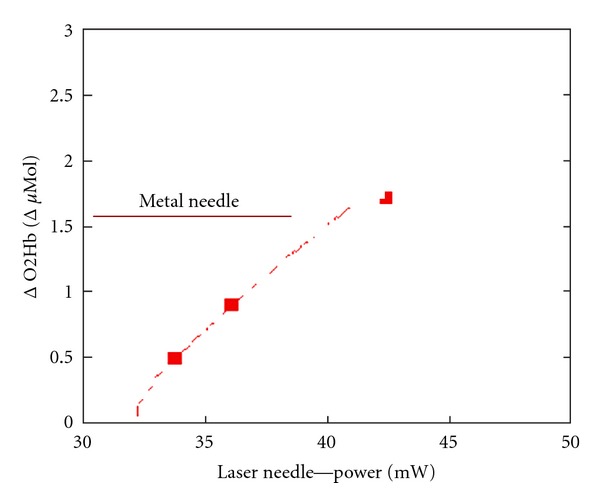
Changes in cerebral oxyhemoglobin concentration using a visual acupuncture scheme with metal needles and laser needles of different optical power. The curve shows the best analytical adaptation to the measurement values of laser needle stimulation (modified from [10]).

Measurements of changes of cerebral concentrations of oxyhemoglobin and deoxyhemoglobin were performed using near infrared spectroscopy (NIRS).  Figure 4 shows one result of these measurements dependent on the optical power of the laser needle stimulation.

## 4. Discussion

Tunér and Hode, both very renowned researchers on laser therapy, stated recently [2]: “anyone who studies the literature carefully can become confused. Some wavelengths achieve the best effects on this and that, while others have poorer effects or none at all. Some doses lead to beneficial effects, but when the dose is increased, the effects wear off. If we treat a condition, some of the parameters we want to influence may be affected, but perhaps not all. If we administer treatment from a distance, we do not get the same effects as if we treat in contact or with pressure. Some frequencies produce effects on pain, others on oedema. What are we to believe? And what do we do to find the best dose, wavelength, and so forth?”

Studies concerning the minimal dose in laser acupuncture are rare. Yurtkuran et al. [11] investigated the effects and minimum effective dose of laser acupuncture in knee osteoarthritis. Patients received 904 nm low-level laser irradiation with 10 mW/cm² power density, 4 mW output power, 0.4 cm² spot size, 0.48 J dose per session, and 120 sec treatment time on the median side of the knee to the Yinlingquan acupuncture point SP9 (Spleen 9). Laser acupuncture, even with this small power density, was found to be effective in reducing periarticular swelling when compared with placebo laser [11]. In a recent Sino-European transcontinental animal experimental study, which was designed by our group and performed at the China Academy of Chinese Medical Sciences in Beijing, we found that ultra-low-level laser acupuncture stimulation in rats can reproducibly induce effects on neurovegetative parameters (see Section 3.1) [8].

Blood flow velocity in the ophthalmic artery in humans is an effective parameter for quantification of the effects of acupuncture treatment and is logarithmically dependent on the stimulus intensity of the laser needles. Thus, we can conclude that Weber-Fechner′s law is valid for the dose-effect relationship examined here. The threshold value for optical power density (I*) can be calculated from the registered and analytically determined effect curve, I* = 1.3 W/cm². This indicates that the optical power density of the laser needles must be greater than 1.3 W/cm² in order to activate the physiological effects of acupuncture. In addition, we can see that the needle equivalence in optical power densities of the laser needles reaches I ≥ 5 W/cm². We can assume that an increase in blood flow velocity in the ophthalmic artery is based on a complex cerebral reaction resulting from acupoint stimulation, preceded by multisynaptic switching of optically induced acupuncture stimulation potentials [6, 10, 12].

It is noteworthy that despite the physiological complexity, the logarithmic relationship between stimulus strength I and stimulus effect is maintained. We interpret this as obvious proof that specific effects of acupuncture underlie these logarithmic dose-effect relationships. The existence and validity of dose-effect relationships in acupuncture could be proven for the first time using the methods described [6, 10]. This statement is strictly valid only when using laser needles which trigger continuous permanent stimulation, thus allowing exact quantification of stimulus strength. To what extent low- or high- frequency modulation of laser needle light can modify proven dose-effect relationships is unclear and must be investigated in further studies. Since the postulated equivalence between metal needles and laser needles could be clearly shown in the examined context, we can conclude that classical acupuncture and its effects also should be functionally dependent on stimulus strength according to a potency rule [6, 10].

Experimental data of our research group in Figure 4 show that laser needle stimulation with an optical power of about 40 mW leads to changes in oxyhemoglobin concentration, similar to the effects when using metal needles. The equivalency between metal needle stimulation and laser needle stimulation can also be proven with these cerebral effects [13]. These experiments also yield the best analytical adaptation of the measurement results in a logarithmic function, that is, cerebral oxyhemoglobin concentration parameters also underlie a physiological dose-effect relationship.

There are several position statements on laser acupuncture [14] and also several review articles in scientific literature concerning clinical effectiveness of laser acupuncture [15]. For example, the Australian Medical Acupuncture College [14] stated that “the optimal energy density for laser acupuncture and biostimulation, based on current clinical experience, is 4 J/cm²”. However, our results of previous research studies [6, 8] and of this publication clearly show that dose must be adjusted according to the individual responses.
